# Communication-Efficient Consensus for Networked Robotic Sensors: A Weighted Sliding Integration-Based Adaptive Dynamic Event-Triggered Approach

**DOI:** 10.3390/s26134006

**Published:** 2026-06-24

**Authors:** Xing Gu, Ning Lin, Bo Li, Zhikang Zhou, Zhicheng Hou

**Affiliations:** 1 School of Mechanical Engineering and Electronic Information, China University of Geosciences, Wuhan 430074, China; 2 College for Elite Engineers, China University of Geosciences, Wuhan 430074, China; 3 Yunfu CAS Stone Innovation Technology Co., Ltd., Yunfu 527300, China; 4 Department of Automation, Guangdong Polytechnic Normal University, Guangzhou 510665, China

**Keywords:** networked robotic sensors, adaptive dynamic event-triggered control, weighted sliding integration

## Abstract

This paper addresses the consensus problem for networked robotic sensors characterized by general linear dynamics and strict communication bandwidth limitations. We propose a weighted sliding integration-based adaptive dynamic event-triggered control (WSI-ADETC) strategy. First, we design a bounded adaptive parameter using a nonlinear protocol to enhance sensitivity to changes in consensus error. To further alleviate the communication burden on the sensing network, we propose a weighted sliding integration-based event-triggering mechanism to reduce the number of triggers compared to traditional adaptive dynamic event-triggered control (ADETC) approaches. Using Lyapunov analysis, we establish sufficient conditions for asymptotic consensus and demonstrate that the proposed controller effectively eliminates Zeno behavior. Numerical simulations demonstrate that the proposed WSI-ADETC strategy significantly reduces communication frequency while maintaining satisfactory consensus performance. Compared with recent adaptive dynamic event-triggered methods, the proposed method reduces the total triggering number by more than 53%, providing a communication efficient solution for resource-constrained robotic sensing networks.

## 1. Introduction

Consensus control of multi-agent systems (MASs) provides the theoretical framework for networked robotic sensors in applications like environmental monitoring and swarm coordination, where the exchange of state information between agents is typically conducted over wireless sensor networks [1]. However, these networks often suffer from severe bandwidth limitations and communication congestion [2]. Traditional control schemes, which require continuous sensor data transmission, lead to an unnecessary waste of limited communication resources.

Therefore, developing a control strategy that maintains consensus while minimizing the sensor-to-controller data rate has practical significance [3]. To this end, event-triggered and event-based control strategies have been extensively investigated since the pioneering studies on event-based sampling and control in the late 20th century [4,5,6], first implemented in MASs [7], and further developed in [8,9,10,11,12,13], etc.

To reduce communication burden, numerous event-triggered consensus protocols have been developed for MASs. However, many existing approaches rely on global information of the communication graph. For example, the smallest non-zero eigenvalue of the Laplacian is used to design the ETM in [14,15] and determine the minimum sampling interval in [16].

However, in practice, obtaining global information is challenging, especially when the scale of MAS is large and the communication is distributed. In order to eliminate the reliance of global information, the adaptive event-triggered control (AETC) has been developed. In AETC, the controller is designed using time-varying gains, governed by some adaptive protocols. Within this framework, fully distributed event-triggered control is first proposed in [17]. The output consensus problem of heterogeneous linear multi-agent systems (MASs) is investigated in [18], while Cheng and Li (2019) [19] focuses on the consensus of MASs with nonidentical uncertainties. Furthermore, Qian et al. (2020) [20] presents an AETC strategy with output feedback to address the consensus problem of linear MASs subjected to external disturbances. The study in [21] examines the consensus of linear MASs on directed graphs, and Wu et al. (2021) [22] explores cooperative tracking control in MASs modeled as a Lur’e system.

Although AETC achieves fully distributed implementation, the communication burden can still be further reduced. To this end, researchers explore the adaptive dynamic ETC (ADETC), which is first detailed in [23]. In [24], ADETC is extended to unknown second-order nonlinear MASs. In recent years, ADETC has attracted considerable attention. For example, He et al. (2020) [25] investigates the consensus of general linear MAS in both leaderless and leader-following networks. Different from [25], the control strategy proposed in [26] is fully distributed. The work in [27] focuses on implementing ADETC for nonlinear MASs, specifically addressing the fault-tolerant consensus problem. More recently, Chen (2024) [28] proposes a triggered jump ADETC (TJADETC) to ensure the hardware response speed required for event-triggered control.

Although ADETC further reduces communication burden by introducing dynamic internal variables into the triggering mechanism, several challenges remain. The designed gains of ADETC are not effectively adaptive to the variation of consensus error, leading to excessively high gains even after the convergence of consensus error. Although the designed adaptive gain in [29] can adapt the variation of consensus error, it is not sufficiently responsive to that variation, due to the inertia of the designed linear adaptive protocol. Furthermore, most of these studies utilize exponential functions as triggering thresholds, which may not be suitable for practical systems with disturbances. In such cases, the triggering frequency tends to increase when the consensus errors nearly converge to zero, since the value of the exponential function approaches zero.

Facing the challenges in achieving responsive adaptive gain regulation and efficient triggering decisions for networked sensing systems under bandwidth constraints, a novel weighted sliding integration-based adaptive dynamic event-triggered control (WSI-ADETC) strategy is proposed in this paper. Although integration-based event-triggered mechanisms have also been seen in the literature, such as in [30,31], the proposed mechanism differs fundamentally from these approaches. The proposed method introduces a weighted sliding integration mechanism within an adaptive dynamic event-triggered consensus framework, instead of using global integration, which has the advantage of forgetting property. By combining instantaneous consensus information with accumulated historical triggering behavior, the proposed mechanism provides a memory effect capable of suppressing unnecessary triggering events caused by transient disturbances.

Compared with existing approaches, the contributions of this work are threefold. First, a bounded nonlinear adaptive protocol is developed to generate adaptive gains that remain responsive to consensus error variations while avoiding excessive gain growth after convergence. Second, a weighted sliding integration-based triggering mechanism is proposed to introduce a memory effect through accumulated historical triggering information. Different from conventional triggering laws relying mainly on instantaneous errors, the proposed mechanism can effectively suppress unnecessary triggering events caused by transient disturbances. Third, rigorous Lyapunov-based analysis is provided to establish asymptotic consensus and Zeno-behavior exclusion for general linear multi-agent systems. Additionally, an exponential function is introduced to ensure the hardware response speed required for event-triggered control.

The rest of the paper is organized as follows. Section 2 provides a brief overview of algebraic graph theory and presents the problem statement. Section 3, Section 4 and Section 5 introduce the weighted sliding integration-based adaptive dynamic event-triggered control in detail. Section 6 and Section 7 present numerical simulations and conclusions, respectively.

## 2. Preliminaries and Problem Statements

### 2.1. Graph Theory

For MASs, the communication topology of a system can be represented by a graph G with three elements, i.e., vertex set V, edge set E and adjacency matrix A. The vertex set V={1,2,…,N} denotes the ensemble of agents indices. The edge set E={(i,j)|i,j∈V} represents the inter-agent communication links, where (i,j) indicates that agent *i* can receive information from agent *j*. The neighbor set of agent *i* is denoted by $N_{i}=\left\{j|\left(i,j\right)\in E\right\}$, with its cardinality represented by $|N_{i}|$. The element $a_{ij}$ of the adjacency matrix $A\in R^{N\times N}$ satisfies $a_{ij}=1$, if (i,j)∈E, otherwise $a_{ij}=0$. The elements of Laplacian $L\in R^{N\times N}$ satisfy that $l_{ij}=-a_{ij}$ and $l_{ii}=\left|N_{i}\right|$. For undirected graphs, the adjacency matrix satisfies $A^{T}=A$, since (j,i)∈E if (i,j)∈E.

### 2.2. Problem Statements

We consider a group of *N* networked robotic sensors, where the motion and internal dynamics of each agent *i* are modeled by the following multi-agent system with general linear systems:

(1) $${\dot{x}}_{i}=Ax_{i}+Bu_{i},\;i=1,2,\ldots ,N$$

where $x_{i}\in R^{n}$ represents the state vector measured by onboard sensors, and $u_{i}\in R^{m}$ denotes the control input. The system matrices $A\in R^{n\times n}$ and $B\in R^{n\times m}$ characterize the inherent physical dynamics of the robotic platforms.

In networked sensing frameworks, each robotic agent must share its state $x_{i}$ with its neighbors to achieve global consensus. However, due to the bandwidth-limited nature of the wireless sensor network, continuous communication of state $x_{i}$ is not feasible. The objective is to design a control protocol $u_{i}$ and a communication-efficient triggering mechanism that ensures state consensus defined as follows.

**Definition 1.**  *The consensus of the MAS is achieved asymptotically, if the states of agents satisfy $\lim_{t\to \infty }∥x_{i}\left(t\right)-x_{j}\left(t\right)∥=0,\forall i,j\in V$ with any initial state values*.

In practical networked systems, the implementation of any event-triggered strategy must account for the physical limitations of the communication hardware and processing units. A critical requirement for the feasibility of such a strategy is the avoidance of the Zeno behavior, which is defined as follows.

**Definition 2.**  *The Zeno behavior does not exist if the triggering instants satisfy $\inf\{t_{{\left(k+1\right)}_{i}}-t_{{\left(k\right)}_{i}}\}=\epsilon ,0<\epsilon <\infty$, where $t_{{\left(k\right)}_{i}}$ represent the k-th triggering instants of agent i*.

Then, the objective of this paper is to propose a novel adaptive dynamic event-triggered control (ETC), with which the MAS (1) can asymptotically achieve consensus and avoid Zeno behavior.

To facilitate subsequent theorem analysis, some assumptions and lemmas are presented as follows.

**Assumption 1.**  *The system (A,B) is stabilizable*.

**Assumption 2.**  *The graph G is undirected and connected*.

**Lemma 1.**  *In an undirected graph, the Laplacian $L\in R^{n\times n}$ is semi-positive definite. Its zero eigenvalue corresponds to the eigenvector $1_{n}$ whose elements are all equal to 1. The minimum non-zero eigenvalue of L is denoted by $\lambda_{2}\left(L\right)$, which satisfies*

$$\lambda_{2}\left(L\right)=\min_{x\neq 0,1^{T}x=0}\frac{x^{T}Lx}{x^{T}x},$$

*where $x\in R^{n}$*.

**Lemma 2**  (Young’s inequality)**.** *For vectors $z,w\in R^{n}$ and ϵ>0, it holds that*

$$z^{T}w\leq \frac{\epsilon }{2}z^{T}z+\frac{1}{2\epsilon }w^{T}w.$$

Compared with the classical ADETC approach [26], where adaptive gains may become excessively large and insensitive to error variations, the proposed method introduces a bounded nonlinear adaptive protocol that maintains responsiveness while avoiding overestimation.

Furthermore, unlike TJADETC [28], which improves triggering efficiency via jump mechanisms, the proposed weighted sliding integration-based triggering introduces a memory effect that accumulates error information over time, effectively reducing unnecessary triggering events.

## 3. Controller Design

In this section, a weighted sliding integration-based adaptive dynamic event-triggered control is proposed.

### 3.1. Consensus Protocol with Adaptive Gains

The controller is designed as follows.

(2) $$\left\{\begin{array}{cc} u_{i} & =-K\sum_{j=1}^{N}a_{ij}\beta_{ij}\left({\hat{x}}_{i}-{\hat{x}}_{j}\right)\\ {\dot{\beta }}_{ij} & =\left\{\begin{array}{cc} k_{ij}a_{ij}{\left({\hat{x}}_{i}-{\hat{x}}_{j}\right)}^{T}F\left({\hat{x}}_{i}-{\hat{x}}_{j}\right), & \beta_{ij}\leq \gamma \\ k_{ij}a_{ij}\left[-\iota_{ij}e^{-{\left({\hat{x}}_{i}-{\hat{x}}_{j}\right)}^{T}F\left({\hat{x}}_{i}-{\hat{x}}_{j}\right)}+{\left({\hat{x}}_{i}-{\hat{x}}_{j}\right)}^{T}F\left({\hat{x}}_{i}-{\hat{x}}_{j}\right)\right], & \beta_{ij}>\gamma \end{array}\right. \end{array}\right.$$

where ${\hat{x}}_{i}$ and ${\hat{x}}_{j}$ represent the estimates of the states of agents *i* and *j*, using the state value at each triggering instant, such that ${\hat{x}}_{i}\left(t\right)=e^{A\left(t-t_{{\left(k\right)}_{i}}\right)}x_{i}\left(t_{{\left(k\right)}_{i}}\right),\forall t\in \left[t_{{\left(k\right)}_{i}},t_{{\left(k+1\right)}_{i}}\right)$ and ${\hat{x}}_{j}\left(t\right)=e^{A\left(t-t_{{\left(k\right)}_{j}}\right)}x_{j}\left(t_{{\left(k\right)}_{j}}\right),\forall t\in \left[t_{{\left(k\right)}_{j}},t_{{\left(k+1\right)}_{j}}\right)$. The parameter $k_{ij}$, $j\in N_{i}$ satisfies $k_{ij}=k_{ji}>0$. Notations ι and γ are positive constants. The feedback matrix *K* is designed as $K=B^{T}P$, the matrix *F* represents matrix $PBB^{T}P$, where *P* represents the solution of the *ARE* (Algebraic Riccati Equation) $PA+A^{T}P+Q-PBB^{T}P=0$ for a given positive-definite matrix *Q*. In this paper, the matrix *Q* is set to be an identity. It is worth noting that the adaptive parameter $\beta_{ij}$ is designed to satisfy $\beta_{ij}=\beta_{ji}>0$.

**Remark 1.**  *The estimated agent states are employed to reduce the measurement error $e_{i}={\hat{x}}_{i}-x_{i}$ between two consecutive triggering instants, particularly when the consensus error is small. If, instead, the states at the triggering instants are used in the controller over the interval between two triggers, the resulting measurement error becomes $e_{i}^{\prime }={\hat{x}}_{i}-x_{i}\left(t_{{\left(k\right)}_{i}}\right)$ for $t\in [t_{{\left(k\right)}_{i}},t_{{\left(k+1\right)}_{i}})$, which is generally larger*.

**Remark 2.**  *According to the design of the adaptive parameter $\beta_{ij}$, it follows that $\beta_{ij}$ converges to γ once consensus among the agents is achieved, where γ is a prescribed constant. In contrast to existing works such as [27,29], in which the adaptive gain may grow excessively large and become unpredictable after consensus is reached, the proposed adaptive gain remains bounded and predictable*.

### 3.2. Weighted Sliding Integration-Based Event-Triggering Mechanism

For each agent, the event-triggering mechanism is designed as follows.

(3) $$f_{i}\geq \mu e^{-\rho_{i}\left(t-t_{{\left(k\right)}_{i}}\right)}+\omega_{i}\theta_{i},\forall i\in V$$

where the parameters are chosen to satisfy μ>0 and $\rho_{i}>0$; the functions are designed as

$$\left\{\begin{array}{c} f_{i}=\sum_{j=1}^{N}\left(1+\beta_{ij}\right)a_{ij}e_{i}^{T}Fe_{i}-\frac{\varrho }{4}\sum_{j=1}^{N}a_{ij}{\left({\hat{x}}_{i}-{\hat{x}}_{j}\right)}^{T}F\left({\hat{x}}_{i}-{\hat{x}}_{j}\right)\\ \theta_{i}=-\int_{t_{k}^{i}}^{t}f_{i}-\mu e^{-\rho_{i}\left(s-t_{{\left(k\right)}_{i}}\right)}ds\\ {\dot{\omega }}_{i}=-\kappa_{i}\omega_{i}+\sigma_{i}\sum_{j=1}^{N}a_{ij}{\left({\hat{x}}_{i}-{\hat{x}}_{j}\right)}^{T}F\left({\hat{x}}_{i}-{\hat{x}}_{j}\right)+c \end{array}\right.$$

where the state measurement error is represented by $e_{i}={\hat{x}}_{i}-x_{i}$. The initial value of $\omega_{i}$ is selected satisfying $\omega_{i}\left(0\right)>0$. The parameters μ, $\rho_{i}$, ϱ, $\kappa_{i}$, $\sigma_{i}$, and *c* are some positive constants.

**Remark 3.**  *The event-triggering mechanism in (3) is constructed by integrating instantaneous error feedback with a dynamic threshold evolution. The function $f_{i}$ captures the current consensus error and measurement mismatch, ensuring that triggering decisions are sensitive to system deviations. The exponential term $\mu e^{-\rho_{i}\left(t-t_{i}^{k}\right)}$ provides a decaying lower bound on the triggering condition. The parameter $\rho_{i}$ governs the exponential decay rate in the triggering threshold, thereby influencing the transient triggering behavior*.

**Remark 4.**  *The term $\omega_{i}\theta_{i}$ introduces a weighted sliding integration effect that accumulates historical information of the triggering function through the online evolution of the internal dynamic variables $\theta_{i}$ and $\omega_{i}$. This design gives the mechanism a memory property, allowing it to filter out high-frequency oscillations and transient disturbances. Consequently, compared with conventional dynamic event-triggered schemes, the proposed mechanism can significantly reduce unnecessary triggering events while preserving convergence performance*.

**Remark 5.**  *The parameters $\kappa_{i}$ and $\sigma_{i}$ regulate the evolution of the internal dynamic variable $\omega_{i}$, which affects the responsiveness and smoothness of the triggering mechanism. The constant c prevents the variable $\omega_{i}$ from decaying to zero*.

## 4. Stability Analysis

Before we discuss the stability of the system, we prove the following proposition.

**Proposition 1.**  *The internal dynamic variable $\theta_{i}\left(t\right)$ in the event-triggering mechanism (3) remains non-negative for all t≥0*.

**Proof.**  Between any two adjacent triggering instants, i.e., for $t\in \left(t_{{\left(k\right)}_{i}},t_{{\left(k+1\right)}_{i}}\right)$, the time derivative of $\theta_{i}\left(t\right)$ is given by ${\dot{\theta }}_{i}=-f_{i}+\mu e^{-\rho_{i}\left(s-t_{{\left(k\right)}_{i}}\right)}$. According to the event-triggered condition (3), it holds that $f_{i}<\mu e^{-\rho_{i}\left(t-t_{{\left(k\right)}_{i}}\right)}+\omega_{i}\theta_{i}$ for all $t\in \left(t_{{\left(k\right)}_{i}},t_{{\left(k+1\right)}_{i}}\right)$, which implies that $-f_{i}+\mu e^{-\rho_{i}\left(t-t_{{\left(k\right)}_{i}}\right)}>-\omega_{i}\theta_{i}$. Therefore, we obtain the differential inequality ${\dot{\theta }}_{i}>-\omega_{i}\theta_{i}$. Based on the design of $\omega_{i}$ in (3) and given the initial condition $\omega_{i}\left(0\right)>0$, we have $\omega_{i}>0$. Note that at the triggering instants $t_{{\left(k\right)}_{i}}$, $\theta_{i}\left(t_{{\left(k\right)}_{i}}\right)=0$; therefore, $\theta_{i}\geq 0$ when $t\in \left[t_{{\left(k\right)}_{i}},t_{{\left(k+1\right)}_{i}}\right)$. Thus, $\theta_{i}\geq 0$ when t∈[0,∞). □

Next, we show that multi-agent systems can achieve asymptotic consensus by using the proposed control protocol.

**Theorem 1.**  *Under Assumptions 1 and 2, the multi-agent system (1) achieves asymptotic consensus under the controller (2) and the event-triggering mechanism (3), provided that the following conditions are satisfied:*

*(i)*  *The control gain is designed as $K=B^{T}P$, where P is the positive definite solution to the Algebraic Riccati Equation (ARE)*
  (4) $$PA+A^{T}P+Q-PBB^{T}P=0$$
  *with a given positive definite matrix Q*.
*(ii)*  *The adaptive parameters satisfy $k_{ij}=k_{ji}>0$, ι>0, γ>0, and $\beta_{ij}\left(0\right)>0$*.
*(iii)*  *The design parameters ϵ and ς satisfy $\epsilon >\frac{1}{2}$ and $\varsigma >\max\{1,\epsilon \lambda_{2}\left(L\right)\}$, and the controller parameter γ in (2) is chosen such that*
  (5) $$\gamma \geq \frac{\varsigma }{\nu \left(\varsigma -1\right)\lambda_{2}\left(L\right)}$$
  *where $\mu +\nu =1-\frac{1}{2\epsilon }$ with μ,ν>0*.
*(iv)*  *The event-triggering parameters μ, $\rho_{i}$, ϱ, $\kappa_{i}$, $\sigma_{i}$, and c are all positive constants, $\varrho =\frac{\mu }{\nu (\varsigma -1)}$ and the initial condition satisfies $\omega_{i}\left(0\right)>0$*.

*Then, the consensus error converges to the origin asymptotically*.

**Proof of Theorem 1.**  Let us design the Lyapunov candidate function as follows.

(6) $$V=V_{1}+V_{2}+V_{3}$$

where

$$\left\{\begin{array}{cc} V_{1} & =\sum_{i=1}^{N}\xi_{i}^{T}P\xi_{i}\\ V_{2} & =\sum_{i=1}^{N}\sum_{j=1}^{N}\frac{\left(2\epsilon -1\right)a_{ij}{\left(\beta_{ij}-\gamma \right)}^{2}}{4\epsilon k_{ij}}\\ V_{3} & =4\nu \left(\varsigma -1\right)\gamma \sum_{i=1}^{N}\left(\theta_{i}+\frac{\mu }{\rho_{i}}e^{-\rho_{i}\left(t-t_{{\left(k\right)}_{i}}\right)}\right) \end{array}\right.$$

The consensus error of agent *i* is defined as $\xi_{i}=x_{i}-\frac{1}{N}\sum_{k=1}^{N}x_{k}$. Note that $\beta_{ij}=\beta_{ji}$, and ${\dot{\xi }}_{i}=A\xi_{i}-BK\sum_{j=1}^{N}\beta_{ij}a_{ij}\left({\hat{x}}_{i}-{\hat{x}}_{j}\right)$; the derivative of $V_{1}$ can be rewritten as follows. By calculating the derivative of $V_{1}$, we can obtain

(7) $${\dot{V}}_{1}=\sum_{i=1}^{N}\xi_{i}^{T}\left(PA+A^{T}P\right)\xi_{i}-\sum_{i=1}^{N}\sum_{j=1}^{N}\beta_{ij}a_{ij}{\left(\xi_{i}-\xi_{j}\right)}^{T}F\left({\hat{x}}_{i}-{\hat{x}}_{j}\right)$$

Since $\xi_{i}-\xi_{j}=x_{i}-x_{j}$, we have

(8) $$\begin{array}{cc} {\dot{V}}_{1}= & \sum_{i=1}^{N}\xi_{i}^{T}\left(PA+A^{T}P\right)\xi_{i}-\sum_{i=1}^{N}\sum_{j=1}^{N}\beta_{ij}a_{ij}{\left({\hat{x}}_{i}-{\hat{x}}_{j}\right)}^{T}F\left({\hat{x}}_{i}-{\hat{x}}_{j}\right)\\ \; & +\sum_{i=1}^{N}\sum_{j=1}^{N}\beta_{ij}a_{ij}{\left(e_{i}-e_{j}\right)}^{T}F\left({\hat{x}}_{i}-{\hat{x}}_{j}\right) \end{array}$$

where we recall that $e_{i}={\hat{x}}_{i}-x_{i}$. By using Young’s inequality in Lemma 2, we can obtain that

(9) $$\begin{array}{cc} {\dot{V}}_{1}\leq & \sum_{i=1}^{N}\xi_{i}^{T}\left(PA+A^{T}P\right)\xi_{i}-\frac{2\epsilon -1}{2\epsilon }\sum_{i=1}^{N}\sum_{j=1}^{N}\beta_{ij}a_{ij}{\left({\hat{x}}_{i}-{\hat{x}}_{j}\right)}^{T}F\left({\hat{x}}_{i}-{\hat{x}}_{j}\right)\\ \; & +\frac{\epsilon }{2}\sum_{i=1}^{N}\sum_{j=1}^{N}\beta_{ij}a_{ij}{\left(e_{i}-e_{j}\right)}^{T}F\left(e_{i}-e_{j}\right) \end{array}$$

where ϵ>1/2.

According to the selection of $V_{2}$, its derivative yields,

(10) $${\dot{V}}_{2}=\left\{\begin{array}{cc} \sum_{i=1}^{N}\sum_{j=1}^{N}\frac{\left(2\epsilon -1\right)a_{ij}\left(\beta_{ij}-\gamma \right)}{2\epsilon }{\left({\hat{x}}_{i}-{\hat{x}}_{j}\right)}^{T}F\left({\hat{x}}_{i}-{\hat{x}}_{j}\right), & \beta_{ij}\leq \gamma \\ \sum_{i=1}^{N}\sum_{j=1}^{N}\frac{\left(2\epsilon -1\right)a_{ij}\left(\beta_{ij}-\gamma \right)}{2\epsilon }\left[{\left({\hat{x}}_{i}-{\hat{x}}_{j}\right)}^{T}F\left({\hat{x}}_{i}-{\hat{x}}_{j}\right)-\iota_{ij}e^{-{\left({\hat{x}}_{i}-{\hat{x}}_{j}\right)}^{T}F\left({\hat{x}}_{i}-{\hat{x}}_{j}\right)}\right], & \beta_{ij}>\gamma \end{array}\right.$$

According to (9) and (10), we calculate ${\dot{V}}_{1}+{\dot{V}}_{2}$ in the following two cases.

(a) When $\beta_{ij}\leq \gamma$.

${\dot{V}}_{1}+{\dot{V}}_{2}$ yields,

(11) $$\begin{array}{cc} {\dot{V}}_{1}+{\dot{V}}_{2}\leq & \sum_{i=1}^{N}\xi_{i}^{T}\left(PA+A^{T}P\right)\xi_{i}+\frac{\epsilon }{2}\sum_{i=1}^{N}\sum_{j=1}^{N}\beta_{ij}a_{ij}{\left(e_{i}-e_{j}\right)}^{T}F\left(e_{i}-e_{j}\right)\\ \; & -\sum_{i=1}^{N}\sum_{j=1}^{N}\frac{\left(2\epsilon -1\right)a_{ij}\gamma }{2\epsilon }{\left({\hat{x}}_{i}-{\hat{x}}_{j}\right)}^{T}F\left({\hat{x}}_{i}-{\hat{x}}_{j}\right) \end{array}$$

Noting that ${\hat{x}}_{i}=e_{i}+x_{i}$ and using Young’s inequality, we have

$$\begin{array}{cc} \sum_{i=1}^{N}\sum_{j=1}^{N}a_{ij}{\left({\hat{x}}_{i}-{\hat{x}}_{j}\right)}^{T}F\left({\hat{x}}_{i}-{\hat{x}}_{j}\right)\geq & \left(1-\frac{1}{\varsigma }\right)\sum_{i=1}^{N}\sum_{j=1}^{N}a_{ij}{\left(x_{i}-x_{j}\right)}^{T}F\left(x_{i}-x_{j}\right)\\ & -\left(\varsigma -1\right)\sum_{i=1}^{N}\sum_{j=1}^{N}a_{ij}{\left(e_{i}-e_{j}\right)}^{T}F\left(e_{i}-e_{j}\right) \end{array}$$

Considering the definition of parameters $\mu +\nu =1-\frac{1}{2\epsilon }$, μ, ν>0, we replace the parameters $1-\frac{1}{2\epsilon }$ by using μ+ν. Then, the derivative of $V_{1}+V_{2}$ yields,

(12) $$\begin{array}{cc} {\dot{V}}_{1}+{\dot{V}}_{2}\leq & \sum_{i=1}^{N}\xi_{i}^{T}\left(PA+A^{T}P\right)\xi_{i}-\mu \sum_{i=1}^{N}\sum_{j=1}^{N}\gamma a_{ij}{\left({\hat{x}}_{i}-{\hat{x}}_{j}\right)}^{T}F\left({\hat{x}}_{i}-{\hat{x}}_{j}\right)\\ \; & +\sum_{i=1}^{N}\sum_{j=1}^{N}\left[\frac{\epsilon }{2}\beta_{ij}+\nu \left(\varsigma -1\right)\gamma \right]a_{ij}{\left(e_{i}-e_{j}\right)}^{T}F\left(e_{i}-e_{j}\right)\\ \; & -\nu \left(1-\frac{1}{\varsigma }\right)\gamma \sum_{i=1}^{N}\sum_{j=1}^{N}a_{ij}{\left(x_{i}-x_{j}\right)}^{T}F\left(x_{i}-x_{j}\right) \end{array}$$

Considering the ARE in (4), ${\dot{V}}_{1}+{\dot{V}}_{2}$ yields,

(13) $$\begin{array}{cc} {\dot{V}}_{1}+{\dot{V}}_{2}\leq & \xi^{T}\left[I_{N}⊗\left(PA+A^{T}P\right)-\nu \left(1-\frac{1}{\varsigma }\right)\gamma L⊗F\right]\xi \\ \; & +4\nu \left(\varsigma -1\right)\gamma \sum_{i=1}^{N}\left[\sum_{j=1}^{N}\left(1+\frac{\epsilon }{8\nu (\varsigma -1)\gamma }\beta_{ij}\right)a_{ij}e_{i}^{T}Fe_{i}\right.\\ \; & \left.-\frac{\mu }{4\nu (\varsigma -1)}\sum_{j=1}^{N}a_{ij}{\left({\hat{x}}_{i}-{\hat{x}}_{j}\right)}^{T}F\left({\hat{x}}_{i}-{\hat{x}}_{j}\right)\right] \end{array}$$

where ς>1, and $\xi ={\left[\xi_{1}^{T},\ldots ,\xi_{N}^{T}\right]}^{T}$.

According to condition (iii), we know that $\gamma \geq \frac{\varsigma }{\nu \left(\varsigma -1\right)\lambda_{2}\left(L\right)}>\frac{\epsilon }{8\nu (\varsigma -1)}$; the derivative of $V_{1}+V_{2}$ yields

(14) $$\begin{array}{cc} {\dot{V}}_{1}+{\dot{V}}_{2}\leq & -\xi^{T}\xi +4\nu \left(\varsigma -1\right)\gamma \sum_{i=1}^{N}\left[\sum_{j=1}^{N}\left(1+\beta_{ij}\right)a_{ij}e_{i}^{T}Fe_{i}\right.\\ \; & \left.-\frac{\varrho }{4}\sum_{j=1}^{N}a_{ij}{\left({\hat{x}}_{i}-{\hat{x}}_{j}\right)}^{T}F\left({\hat{x}}_{i}-{\hat{x}}_{j}\right)\right] \end{array}$$

where $\varrho =\frac{\mu }{\nu (\varsigma -1)}$. Noting that with the settings of parameters ς>1, $\mu +\nu =1-\frac{1}{2\epsilon },\left(\mu ,\nu >0\right)$, and ϵ>1/2, we can obtain ϱ>0.

According to the design of ETM (3), the derivative of $V_{3}$ is calculated as follows.

(15) $$\begin{array}{c} {\dot{V}}_{3}=4\nu \left(\varsigma -1\right)\gamma \sum_{i=1}^{N}\left[\frac{\varrho }{4}\sum_{j=1}^{N}a_{ij}{\left({\hat{x}}_{i}-{\hat{x}}_{j}\right)}^{T}F\left({\hat{x}}_{i}-{\hat{x}}_{j}\right)-\sum_{j=1}^{N}\left(1+\beta_{ij}\right)a_{ij}e_{i}^{T}Fe_{i}\right] \end{array}$$

Combining (14) and (15), the derivative of $V_{1}+V_{2}+V_{3}$ yields

(16) $$\begin{array}{cc} {\dot{V}}_{1}+{\dot{V}}_{2}+{\dot{V}}_{3}\leq & -\xi^{T}\xi . \end{array}$$

According to (16), we conclude that the consensus error ξ converges to the origin asymptotically when $\beta_{ij}\leq \gamma$.

(b) When $\beta_{ij}>\gamma$.

(17) $$\begin{array}{cc} {\dot{V}}_{1}+{\dot{V}}_{2}\leq & \sum_{i=1}^{N}\xi_{i}^{T}\left(PA+A^{T}P\right)\xi_{i}+\frac{\epsilon }{2}\sum_{i=1}^{N}\sum_{j=1}^{N}\beta_{ij}a_{ij}{\left(e_{i}-e_{j}\right)}^{T}F\left(e_{i}-e_{j}\right)\\ \; & -\sum_{i=1}^{N}\sum_{j=1}^{N}\frac{\left(2\epsilon -1\right)a_{ij}\gamma }{2\epsilon }{\left({\hat{x}}_{i}-{\hat{x}}_{j}\right)}^{T}F\left({\hat{x}}_{i}-{\hat{x}}_{j}\right)\\ \; & -\sum_{i=1}^{N}\sum_{j=1}^{N}\frac{\left(2\epsilon -1\right)a_{ij}\left(\beta_{ij}-\gamma \right)}{2\epsilon }\iota_{ij}e^{-{\left({\hat{x}}_{i}-{\hat{x}}_{j}\right)}^{T}F\left({\hat{x}}_{i}-{\hat{x}}_{j}\right)} \end{array}$$

Using the ARE in (4), ${\dot{V}}_{1}+{\dot{V}}_{2}$ yields,

(18) $$\begin{array}{cc} {\dot{V}}_{1}+{\dot{V}}_{2}\leq & \xi^{T}\left[I_{N}⊗\left(PA+A^{T}P\right)-\nu \left(1-\frac{1}{\varsigma }\right)\gamma L⊗F\right]\xi \\ \; & +4\nu \left(\varsigma -1\right)\gamma \sum_{i=1}^{N}\left[\sum_{j=1}^{N}\left(1+\frac{\epsilon }{8\nu (\varsigma -1)\gamma }\beta_{ij}\right)a_{ij}e_{i}^{T}Fe_{i}\right.\\ \; & \left.-\frac{\mu }{4\nu (\varsigma -1)}\sum_{j=1}^{N}a_{ij}{\left({\hat{x}}_{i}-{\hat{x}}_{j}\right)}^{T}F\left({\hat{x}}_{i}-{\hat{x}}_{j}\right)\right]\\ \; & -\sum_{i=1}^{N}\sum_{j=1}^{N}\frac{\left(2\epsilon -1\right)a_{ij}\left(\beta_{ij}-\gamma \right)}{2\epsilon }\iota_{ij}e^{-{\left({\hat{x}}_{i}-{\hat{x}}_{j}\right)}^{T}F\left({\hat{x}}_{i}-{\hat{x}}_{j}\right)} \end{array}$$

Similar to the derivations from (13) to (16), the derivative of the Lyapunov function candidate $\dot{V}$ yields,

(19) $$\begin{array}{cc} \dot{V}={\dot{V}}_{1}+{\dot{V}}_{2}+{\dot{V}}_{3}\leq & -\xi^{T}\xi -\sum_{i=1}^{N}\sum_{j=1}^{N}\frac{\left(2\epsilon -1\right)a_{ij}\left(\beta_{ij}-\gamma \right)}{2\epsilon }\iota_{ij}e^{-{\left({\hat{x}}_{i}-{\hat{x}}_{j}\right)}^{T}F\left({\hat{x}}_{i}-{\hat{x}}_{j}\right)}. \end{array}$$

The second term of the right-hand side of (19) $\sum_{i=1}^{N}\sum_{j=1}^{N}\frac{\left(2\epsilon -1\right)a_{ij}\left(\beta_{ij}-\gamma \right)}{2\epsilon }\iota_{ij}e^{-{\left({\hat{x}}_{i}-{\hat{x}}_{j}\right)}^{T}F\left({\hat{x}}_{i}-{\hat{x}}_{j}\right)}$ is non-negative, since $\beta_{ij}>\gamma$ and ϵ>1/2. Thus, we conclude that $\dot{V}$ is negative definite with respect to ξ; therefore, the consensus error ξ converges to the origin asymptotically.

Then, we can obtain $\dot{V}\leq 0$ in both cases. In particular, $\dot{V}\to 0$ if and only if the consensus error ξ→0 and $\beta_{ij}\to \gamma$. Hence, we conclude the states of agents consensus asymptotically by using the event-triggered controller (2) with ETM (3). That completes the proof. □

**Remark 6.**  *The parameters μ and ϱ play both roles in the Lyapunov-based stability analysis and triggering mechanisms. Specifically, the parameter ϱ, defined as $\varrho =\frac{\mu }{\nu (\varsigma -1)}$, is introduced to establish a coupling between the derivative of $V_{1}+V_{2}$ and $V_{3}$. This design enables the cancellation of the error-related terms in (14) and (15), which is essential for guaranteeing the negative definiteness of the Lyapunov derivative. In addition, μ contributes to the decomposition $\mu +\nu =1-\frac{1}{2\epsilon }$ used in the Lyapunov analysis, and it also provides a strictly positive lower bound in the triggering function. This property is critical for excluding Zeno behavior, as shown in Theorem 2*.

## 5. Zeno Behavior Exclusion

In the sequel, we prove that the Zeno behavior is excluded by using the controller (2) with the event-triggering mechanism (3).

**Theorem 2.**  *Under controller (2) with event-triggering mechanism (3), the triggering instants of agent i∈V satisfy $\inf\left\{t_{{\left(k+1\right)}_{i}}-t_{{\left(k\right)}_{i}}\right\}=\epsilon$, where 0<ϵ<∞*.

**Proof of Theorem 2.**  Calculating the derivative of $\frac{1}{2}e_{i}^{T}e_{i}$, we can obtain

(20) $$\frac{1}{2}\frac{de_{i}^{T}e_{i}}{dt}=e_{i}^{T}{\dot{e}}_{i}=∥e_{i}^{T}{\dot{e}}_{i}∥\leq ∥e_{i}∥∥{\dot{e}}_{i}∥$$

Since $\frac{1}{2}e_{i}^{T}e_{i}=\frac{1}{2}{∥e_{i}∥}^{2}$, then the derivative of $\frac{1}{2}e_{i}^{T}e_{i}$ can be written as

(21) $$\frac{1}{2}\frac{de_{i}^{T}e_{i}}{dt}=\frac{1}{2}\frac{d∥e_{i}{∥}^{2}}{dt}=∥e_{i}∥\frac{d∥e_{i}∥}{dt}$$

Substituting (21) into (20), we have

(22) $$\begin{array}{cc} \frac{d∥e_{i}∥}{dt}\leq & ∥{\dot{e}}_{i}∥\\ \leq & ∥A{\hat{x}}_{i}-\left(Ax_{i}-\sum_{j=1}^{N}\beta_{ij}a_{ij}BK\left({\hat{x}}_{i}-{\hat{x}}_{j}\right)\right)∥\\ \leq & ∥Ae_{i}∥+∥\sum_{j=1}^{N}\beta_{ij}a_{ij}BK\left({\hat{x}}_{i}-{\hat{x}}_{j}\right)∥ \end{array}$$

where $t\in (t_{{\left(k\right)}_{i}},t_{{\left(k+1\right)}_{i}})$.

According to Definition 1, if consensus has not yet been achieved, it holds that $\frac{\varrho }{4}\sum_{j=1}^{N}a_{ij}{\left({\hat{x}}_{i}-{\hat{x}}_{j}\right)}^{T}F\left({\hat{x}}_{i}-{\hat{x}}_{j}\right)>0$. When the triggering condition is satisfied, the measurement error $e_{i}={\hat{x}}_{i}-x_{i}$ is immediately forced to zero. Consequently, $f_{i}$ becomes negative, meaning that the triggering condition (3) is no longer satisfied (knowing that the right-hand side of (3) is always positive according to Proposition 1). Therefore, Zeno behavior cannot occur in this case.

If the consensus of MAS is achieved, we can conclude that ${\hat{x}}_{i}={\hat{x}}_{j}$. Hence, for any given finite initial conditions $x_{i}\left(0\right)$, there is a positive constant $\bar{u}$ such that $∥\sum_{j=1}^{N}a_{ij}BK\left({\hat{x}}_{i}-{\hat{x}}_{j}\right)∥\leq \bar{u}$. According to the design of $\beta_{ij}$ in (2), there exist positive constant $\bar{\beta }$ such that $\beta_{ij}\leq \bar{\beta }$. Then, we can rewrite (22) as follows.

(23) $$\begin{array}{c} \frac{d∥e_{i}∥}{dt}-∥A∥∥e_{i}∥\leq \bar{\beta }\bar{u} \end{array}$$

According the comparison principle, we can obtain

(24) $$\left\{\begin{array}{cc} ∥e_{i}\left(t\right)∥\leq \frac{\bar{\beta }\bar{u}}{∥A∥}\left(e^{∥A∥\left(t-t_{{\left(k\right)}_{i}}\right)}-1\right), & ∥A∥\neq 0\\ ∥e_{i}\left(t\right)∥\leq \bar{\beta }\bar{u}\left(t-t_{{\left(k\right)}_{i}}\right), & ∥A∥=0 \end{array}\right.$$

where $t\in (t_{{\left(k\right)}_{i}},t_{{\left(k+1\right)}_{i}})$.

We carry out the proof by using contradiction. Supposing that the Zeno behavior exists; according to Definition 2, we have $t_{{\left(k\right)}_{i}}=t_{{\left(k+1\right)}_{i}}$.

Between the *k*-th and the k+1-th events, considering the inequalities (24), we can obtain

(25) $$∥e_{i}{\left(t\right)}^{T}PB∥=∥e_{i}{\left(t_{{\left(k+1\right)}_{i}}\right)}^{T}PB∥\leq 0$$

The event-triggering condition (3) yields,

(26) $$\begin{array}{c} f_{i}\left(t\right)=f_{i}\left(t_{{\left(k+1\right)}_{i}}\right)\geq \mu e^{-\rho_{i}\left(t_{{\left(k+1\right)}_{i}}-t_{{\left(k\right)}_{i}}\right)}+\omega_{i}\theta_{i}. \end{array}$$

Since the function $\theta_{i}$ is designed as $\theta_{i}=-\int_{t_{{\left(k\right)}_{i}}}^{t}f_{i}-\mu e^{-\rho_{i}\left(s-t_{{\left(k\right)}_{i}}\right)}ds$, we have

(27) $$\begin{array}{cc} \; & \sum_{j=1}^{N}\left(1+\beta_{ij}\right)a_{ij}e_{i}^{T}Fe_{i}-\frac{\varrho }{4}\sum_{j=1}^{N}a_{ij}{\left({\hat{x}}_{i}-{\hat{x}}_{j}\right)}^{T}F\left({\hat{x}}_{i}-{\hat{x}}_{j}\right)\geq \mu \end{array}$$

where the parameter μ satisfies μ>0. According to the inequalities (27), we can further obtain

(28) $$\begin{array}{c} ∥e_{i}{\left(t\right)}^{T}PB∥\geq \sqrt{\frac{\frac{\varrho }{4}\sum_{j=1}^{N}a_{ij}{∥{\left({\hat{x}}_{i}-{\hat{x}}_{j}\right)}^{T}PB∥}^{2}+\mu }{|N_{i}|\left(1+\bar{\beta }\right)}}>0 \end{array}$$

We observe that (28) contradicts (25), which allows us to conclude that, under the conditions of Theorem 1, the Zeno behavior does not occur when using the controller (2) with the event-triggering mechanism (3). Thus, the proof is complete. □

## 6. Simulation

**Figure 1:**
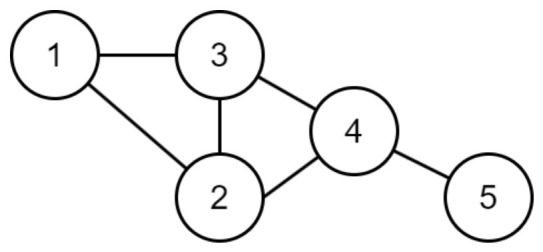
The communication topology of MAS consisting of five agents.

To validate the effectiveness of the proposed WSI-ADETC strategy, we consider a group of five networked robotic sensors tasked with reaching a common consensus state. Each agent is equipped with onboard sensing and wireless communication modules, where the objective is to coordinate their states while strictly minimizing the utilization of the shared communication bandwidth. The communication topology of this sensing network is represented by the undirected graph shown in Figure 1.

The numerical simulations are carried out using MATLAB R2022b. The simulation is carried out on a computer with an Intel i5-12500 processor, 16 GB memory, a NVIDIAGTX1660SUPER display adapter, and Windows 11 operating system, in which the relative tolerance of the solver is set to 1e-6.

For the agent model, the dynamics matrices are given by A=[0,1,0;0,0,1;0,0,0], B=[0;0;1]. In the ARE, we choose identity matrix *I* as matrix *Q*. By solving the ARE, we can obtain the gain matrices $K=B^{T}P=\left[1.000,2.4142,2.4142\right]$, $F=PBB^{T}P=\left[1.000,2.4142,2.4142;2.4142,5.8284,5.8284;2.4142,5.8284,5.8284\right]$.

To demonstrate the effectiveness and communication efficiency of the proposed WSI-ADETC strategy, comparisons are conducted with several representative event-triggered control methods, including a classical static ETC (SETC) scheme, a recent prescribed-time ETC (PTETC) strategy [8], the adaptive dynamic ETC (ADETC) proposed in [26], and the triggered jump adaptive dynamic ETC (TJADETC) proposed in [28]. The parameters of these methods are chosen as follows.

In the SETC scheme, the triggering condition is constructed directly based on instantaneous state errors without introducing adaptive gains or dynamic threshold variables.

In the PTETC method proposed in [8], the control parameters are selected according to the settings in the original work and the decay rate of $e^{-\rho_{i}t}$ is designed by $\rho_{i}=0.5$ to guarantee consensus performance and reduce the triggering number at the same time.

In the ADETC (proposed in [26]), the initial adaptive parameter of the controller is set to $\alpha_{j}\left(0\right)=1$. In the event-triggering mechanism, the parameters are chosen as $\theta_{j}=1$, $\rho_{j}=0.5$, $\sigma_{j}=0.8$, and the initial value of the triggering threshold is set to $\delta_{j}\left(0\right)=1$.

In the TJADETC (proposed in [28]), the parameters of the controller are given by ${\tilde{\kappa }}_{i}=5\times 10^{-3}$, ${\bar{\varrho }}_{i}=1+0.1\times i$, where i=1,2,…,5. The initial value of the adaptive parameter is set to $\varrho_{i}\left(0\right)=0.1$. In the event-triggering mechanism, the parameters are chosen as $\sigma_{i}=10^{0.5}$, ${\tilde{\beta }}_{i}=-10^{-6}$. The initial value of $\phi_{i}$ is set to $\phi_{i}\left(0\right)=0.1$ and the value of the event-triggering function is reset to ${\tilde{\psi }}_{i}\left(t_{k}^{i}\right)=1$ if the event-triggering condition is satisfied.

In our proposed WSI-ADETC, the parameters of the controller (2) are chosen as $k_{ij}=0.2$, ι=1.5, γ=0.8, where the initial value of the adaptive parameter is set to $\beta_{ij}\left(0\right)=1$. In the event-triggering mechanism (3), the parameters are given by $\mu =1\times 10^{-3}$, $\rho_{i}=0.5$, ϱ=1. In addition, the other parameters are given by $\kappa_{i}=1$, $\sigma_{i}=1$, c=1, and the initial value of $\omega_{i}$ is set to $\omega_{i}\left(0\right)=1$.

We assume that the states of agents 1,2,3 are disturbed by pulses at 23 s, 22 s, and 24 s, respectively. The width of pulses is 2 s and the magnitude is 3.

**Figure 2:**
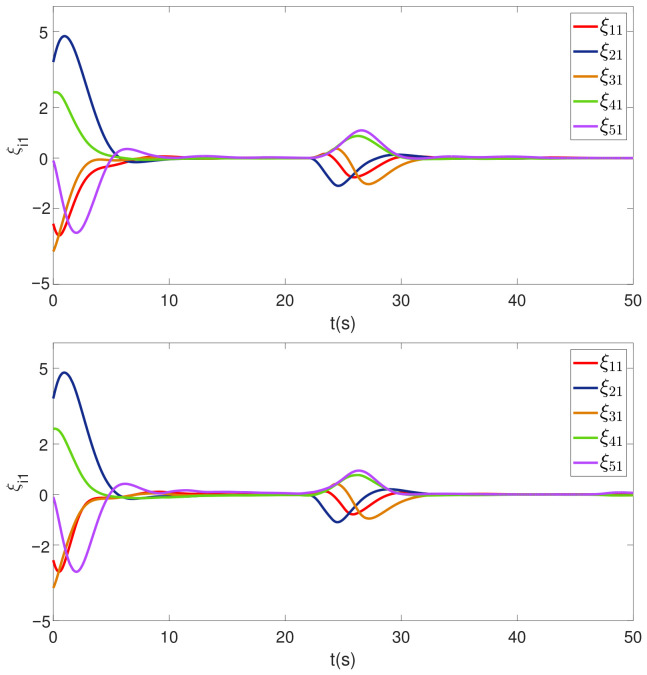
Consensus errors of agents using the SETC (**top**), and PTETC (proposed in [8]) (**bottom**). The states of agent 1,2,3 are disturbed by pulses at 23 s, 22 s, and 24 s, respectively, each with a width of 2 s and a magnitude of 3.

**Figure 3:**
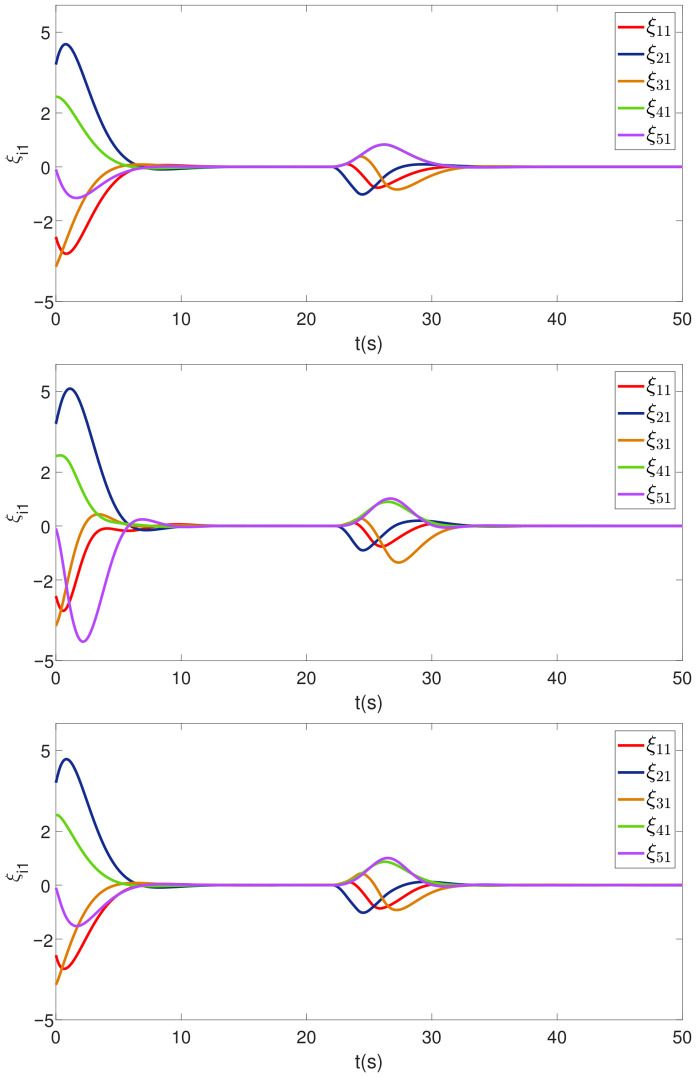
Consensus errors of agents using the ADETC (proposed in [26]) (**top**), TJADETC (proposed in [28]) (**middle**), and the WSI-ADETC (**bottom**). The states of agent 1,2,3 are disturbed by pulses at 23 s, 22 s, and 24 s, respectively, each with a width of 2 s and a magnitude of 3.

The consensus error of the five agents under the SETC and PTETC methods are presented in Figure 2, while Figure 3 shows the comparison of consensus errors by using controllers ADETC, TJADETC, and our controller WSI-ADETC.

Compared with the SETC and PTETC methods, the adaptive dynamic triggering mechanisms significantly reduce the communication burden. It can be observed that both methods can also achieve consensus under the considered disturbance condition. However, compared with the proposed WSI-ADETC strategy, these methods require substantially more triggering events, as shown in lines 1, 2 and 5 in Table 1. In addition, the consensus error is larger than WSI-ADETC (on the bottom of Figure 3).

We can observe that both the ADETC (on the top of Figure 3) and our controller (on the bottom of Figure 3) show faster convergence speed of consensus errors than TJADETC. Comparing the top and bottom subfigures, the convergence speed of consensus errors by using the proposed controller is similar to that using the ADETC.

**Figure 4:**
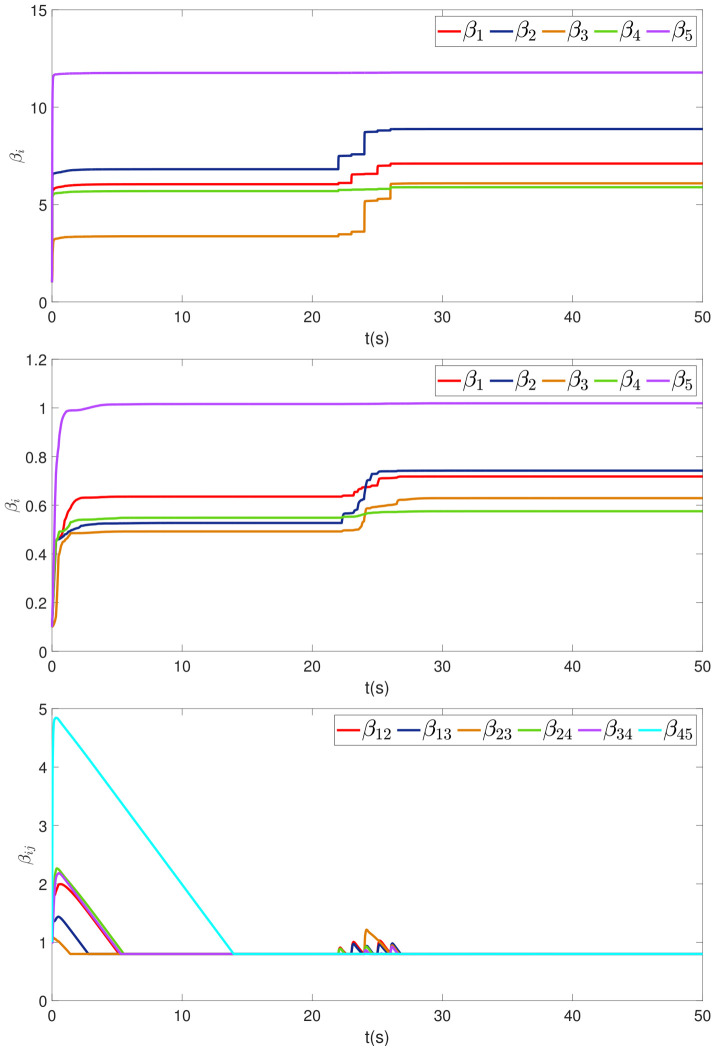
The value of the adaptive gains using the ADETC (proposed in [26]) (**top**), TJADETC (proposed in [28]) (**middle**), and the WSI-ADETC (**bottom**). The states of agent 1,2,3 are disturbed by pulses at 23 s, 22 s, and 24 s, respectively, each with a width of 2 s and a magnitude of 3.

Figure 4 shows the comparison of adaptive gains under ADETC, TJADETC, and our controller WSI-ADETC. From the top and middle subfigures, we can observe that the values of adaptive gains using the ADETC and TJADETC increase to a large value. Then, they remain unchanged after the convergence of consensus errors. Thus, the adaptive parameters cannot adapt to the change of consensus errors. In contrast to the ADETC and TJADETC, the adaptive parameter’s value can adapt to the changes of consensus errors by using the proposed WSI-ADETC.

**Figure 5:**
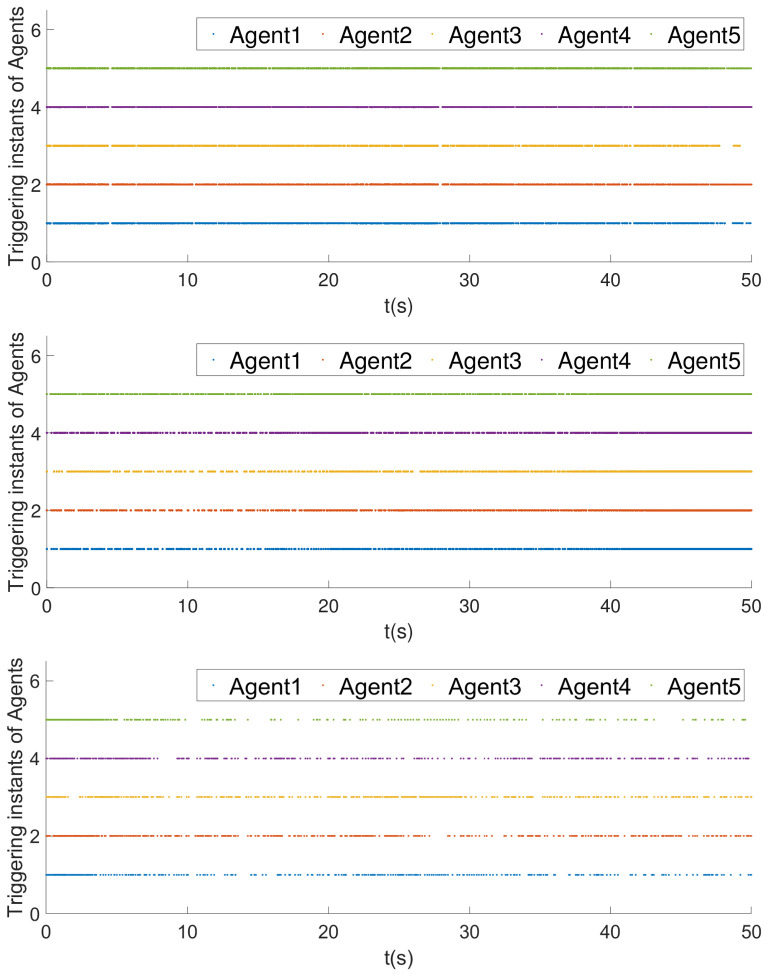
Comparisons of triggering number of agents, by using the ADETC (proposed in [26]) (**top**), TJADETC (proposed in [28]) (**middle**), and the WSI-ADETC (**bottom**). The states of agent 1,2,3 are disturbed by pulses at 23 s, 22 s, and 24 s, respectively, each with a width of 2 s and a magnitude of 3.

The comparisons of triggering number under ADETC, TJADETC, and our controller WSI-ADETC are shown in Figure 5. Comparing the top and middle subfigures, we can observe that the triggering number using TJADETC is much fewer than that of ADETC. In addition, comparing the middle and bottom subfigures, it is evident that our proposed WSI-ADETC results in a much lower triggering number than TJADETC. This result can also be observed from Table 1.

The comparisons of triggering numbers are shown in Table 1. It can be observed that the classical SETC produces the largest number of triggering events, since the triggering condition is constructed directly from instantaneous state errors without adaptive adjustment or dynamic threshold evolution. The proposed WSI-ADETC achieves the smallest triggering number among all compared methods.

Compared with ADETC and TJADETC, the proposed weighted sliding integration-based triggering mechanism further suppresses unnecessary triggering events by introducing a memory effect through the weighted sliding integration term. Consequently, the proposed method achieves substantially improved communication efficiency (observed from the last three lines in Table 1 and Figure 5) while maintaining satisfactory consensus performance (observed from Figure 3).

**Figure 6:**
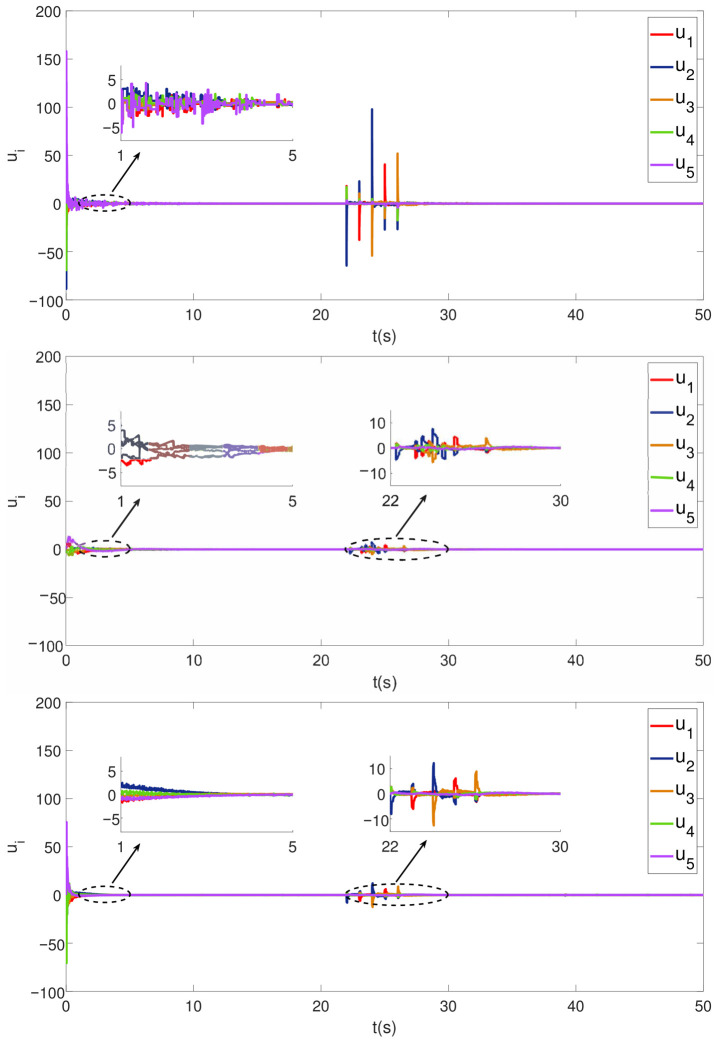
Comparisons of control outputs of agents, by using the ADETC (proposed in [26]) (**top**), TJADETC (proposed in [28]) (**middle**), and the WSI-ADETC (**bottom**). The states of agent 1,2,3 are disturbed by pulses at 23 s, 22 s, and 24 s, respectively, each with a width of 2 s and a magnitude of 3.

The control outputs under ADETC, TJADETC, and WSI-ADETC are compared in Figure 6. We can observe that the controller in ADETC (on the top of the figure) has a larger magnitude of control outputs than the other two controllers. The magnitude of our control outputs (on the bottom of the figure) is smoother than that of TJADETC.

## 7. Conclusions and Discussions

### 7.1. Conclusions

In this paper, we propose a weighted sliding integration-based adaptive dynamic event-triggered control (WSI-ADETC) strategy designed for networked robotic sensors. By integrating a bounded nonlinear adaptive protocol with a sliding integration-based triggering law, the sensor data communication can be reduced. The stability of the system and the achievement of asymptotic consensus for general linear agent dynamics have been rigorously verified through Lyapunov theoretical analysis. Furthermore, we mathematically demonstrate that the proposed strategy eliminates Zeno behavior, ensuring the physical realizability of the controller on a real robotic sensing network. Numerical simulations involving a group of five robotic agents under pulse disturbances have highlighted the advantages of our approach. Specifically, the WSI-ADETC strategy achieves a significant reduction in the total number of data transmissions, outperforming recent adaptive dynamic event-triggered methods by over 50%, while maintaining comparable convergence speeds and smoother control outputs. These results confirm that the proposed framework provides a robust and communication-efficient solution for resource-constrained robotic sensing networks.

### 7.2. Discussions

In this paper, the communication topology is assumed to be undirected and connected, which is commonly adopted in distributed control of multi-agent systems and is practically feasible in many wireless sensor networks with bidirectional communication. The extension of the proposed method to more general cases, such as directed or switching graphs and large-scale networked sensing systems, is an interesting topic and will be considered in future work.

## Figures and Tables

**Table 1:** Comparisons of triggering number of five agents with event-triggered controllers.

Agent Indices	1	2	3	4	5	Total
SETC	5384	5182	5191	4921	4480	25,158
PTETC [8]	4412	4448	4396	4499	3877	21,632
ADETC [26]	1385	1590	1326	1785	1580	7666
TJADETC [28]	847	1040	904	885	656	4332
WSI-ADETC	337	501	416	403	368	2025

## Data Availability

The data presented in this study are available on request from the corresponding author.
